# Smart Poly(lactide)-*b*-poly(triethylene glycol methyl ether methacrylate) (PLA-*b*-PTEGMA) Block Copolymers: One-Pot Synthesis, Temperature Behavior, and Controlled Release of Paclitaxel

**DOI:** 10.3390/pharmaceutics15041191

**Published:** 2023-04-08

**Authors:** Svetlana Lukáš Petrova, Martina Vragović, Ewa Pavlova, Zulfiya Černochová, Alessandro Jäger, Eliézer Jäger, Rafał Konefał

**Affiliations:** Institute of Macromolecular Chemistry CAS, Heyrovského nám. 2, 162 06 Prague, Czech Republic; petrova@imc.cas.cz (S.L.P.); vragovic@imc.cas.cz (M.V.); pavlova@imc.cas.cz (E.P.); cernochova@imc.cas.cz (Z.Č.); alejager@gmail.com (A.J.); jager@imc.cas.cz (E.J.)

**Keywords:** TEGMA, PLA, DLS, NMR, NOESY, LCST, drug release, thermo-responsive polymer, RAFT-ROP

## Abstract

This paper introduces a new class of amphiphilic block copolymers created by combining two polymers: polylactic acid (PLA), a biocompatible and biodegradable hydrophobic polyester used for cargo encapsulation, and a hydrophilic polymer composed of oligo ethylene glycol chains (triethylene glycol methyl ether methacrylate, TEGMA), which provides stability and repellent properties with added thermo-responsiveness. The PLA-*b*-PTEGMA block copolymers were synthesized using ring-opening polymerization (ROP) and reversible addition–fragmentation chain transfer (RAFT) polymerization (ROP-RAFT), resulting in varying ratios between the hydrophobic and hydrophilic blocks. Standard techniques, such as size exclusion chromatography (SEC) and ^1^H NMR spectroscopy, were used to characterize the block copolymers, while ^1^H NMR spectroscopy, 2D nuclear Overhauser effect spectroscopy (NOESY), and dynamic light scattering (DLS) were used to analyze the effect of the hydrophobic PLA block on the LCST of the PTEGMA block in aqueous solutions. The results show that the LCST values for the block copolymers decreased with increasing PLA content in the copolymer. The selected block copolymer presented LCST transitions at physiologically relevant temperatures, making it suitable for manufacturing nanoparticles (NPs) and drug encapsulation-release of the chemotherapeutic paclitaxel (PTX) via temperature-triggered drug release mechanism. The drug release profile was found to be temperature-dependent, with PTX release being sustained at all tested conditions, but substantially accelerated at 37 and 40 °C compared to 25 °C. The NPs were stable under simulated physiological conditions. These findings demonstrate that the addition of hydrophobic monomers, such as PLA, can tune the LCST temperatures of thermo-responsive polymers, and that PLA-*b*-PTEGMA copolymers have great potential for use in drug and gene delivery systems via temperature-triggered drug release mechanisms in biomedicine applications.

## 1. Introduction

Stimuli-responsive polymers, also referred to as “smart polymers”, are extensively studied in the field of polymer biomaterials [1,2,3]. Basically, after the application of external stimuli, such as changes in temperature, pH, magnetic field, ionic strength, light irradiation, and ultrasound, or by the addition of enzymes, glucose, and reactive oxygen species (ROS), they significantly change their physical or chemical properties [3,4,5,6,7,8,9,10]. Among the aforementioned stimuli, one of the most extensively investigated are the thermoresponsive polymers. The noninvasive nature of these materials, together with a wide range of uses, particularly in biomedicine, such as in drug/gene delivery cargo, tissue engineering, imaging agents and biosensors, make them very popular [6,11,12,13,14,15,16,17]. In the area of thermo-responsive polymer biomaterials, the greatest amount of attention is focused on polymer systems that exhibit phase transition in water with a lower critical solution temperature (LCST), around 37 °C (physiological body temperature) [18]. These polymers are very soluble (hydrophilic) at room temperature and, at this stage, from a molecular point of view, their chains occur in a random coil conformation. The increase in temperature above LCST (i.e., when introduced into the body) results in the collapse of the polymer chains, which forms a compact globule (an insoluble hydrophobic form of the particles). For drug delivery purposes, these polymers also must be biocompatible and nontoxic. The most widely studied and famous polymer of this type is poly(*N*-isopropylacrylamide) (PNIPAm) with LCST around 32 °C [19,20,21,22]. Over the past years, polymer chemists reported many PNIPAm alternatives, such as poly(2-oxazoline)s [23,24], poly(methyl vinyl ether) (PMVE), and many others [25]. In addition, another group of such polymers receiving a great deal of interest, due to their biocompatibility and nontoxic properties, is the polymethacrylates group, containing short oligo(ethylene glycol) (OEG) side chains. An important feature of these polymers that their LCST depends explicitly on the length of the OEG side chains. Thus, increasing the length of the side chain results in an increase in the LCST of the polymer. For instance, poly(diethylene glycol methyl ether methacrylate) (PDEGMA) with 2 OEG units, poly(triethylene glycol methyl ether methacrylate) (PTEGMA) with 3 OEG units, and OEG chains with 4–9 ethylene glycol units (POEGMA) show LCST around 30 °C, 50 °C, and 70 °C, respectively. Furthermore, a great advantage of such polymers is that their LCST can be tuned according to their application requirements. For example, it has been shown that LCST values close to physiological temperatures (i.e., 310 K, ~37 °C) could be achieved by the copolymerization of OEGMA with various hydrophobic monomers [26,27,28,29,30,31,32]. Of the abovementioned polymethacrylates, PTEGMA appears to be a suitable candidate for biomedical applications because its LCST of ~50 °C can be also tuned by copolymerization with hydrophobic monomers; however, its copolymerization and behavior under temperature variations has seldom been explored.

Usually, characterization studies of temperature-induced behavior of polymer solutions in LCST-type phase separation are provided by the combination of two or more techniques, such as cloud point measurement, dynamic light scattering (DLS), infrared (IR) spectroscopy, differential scanning calorimetry (DSC), NMR spectroscopy, small angle X-ray scattering, etc. [33,34,35,36,37,38,39,40]. Among these methods, DLS and NMR spectroscopy have advantages, because DLS can easily observe temperature induced transitions in the colloidal level and is largely utilized for comparisons, whereas NMR provides quantitative information on the molecular level related to polymer behavior during LCST phase separation. Moreover, 2D ^1^H–^1^H NOESY spectra help to clarify the conformational behavior of macromolecules in solution [22,41,42,43,44,45]; therefore, the combination of DLS and NMR techniques can provide a comprehensive and detailed characterization of phase transitions.

In this report, we present the first series of block copolymers prepared by the combination of two relevant polymers: (1) the hydrophobic polylactic acid (PLA), which is a biocompatible and biodegradable polyester approved by the FDA for the human use in a myriad of biomedical applications and (2) a hydrophilic polymer comprising OEG chains that provide stability and repellent characteristics, with the addition of thermo-responsiveness, constituting a straightforward alternative to polyethylene oxide (PEO). These copolymers were prepared via the elegant combination of ring-opening polymerization (ROP) and reversible addition–fragmentation chain transfer (RAFT) polymerization (ROP-RAFT) with different ratios between the hydrophobic and hydrophilic blocks. For the block copolymer characterization, we applied size exclusion chromatography (SEC) and ^1^H NMR spectroscopy, whereas ^1^H NMR spectroscopy and 2D nuclear Overhauser effect spectroscopy (NOESY) in combination with dynamic light scattering (DLS) was utilized to comprehensively study and characterize the effect of the hydrophobic PLA block on the LCST of the PTEGMA block in aqueous solutions. From the several PLA-*b*-PTEGMA block copolymers studied, we selected the one which presented LCST transitions at relevant temperatures; more specifically, in hyperthermia treatment (38–45 °C) [46,47], as this can be selectively activated at the tumor sites for drug encapsulation and release of the cargo content. Altogether, we demonstrate that PLA-*b*-TEGMA copolymers are suitable candidates for applications in biomedicine and/or drug and gene delivery systems.

## 2. Materials and Methods

### 2.1. Materials

We recrystallized 3,6-Dimethyl-1,4-dioxane-2,5-dione (lactide, LA, 99%) a few times from ethyl acetate prior to use. Triethylene glycol methyl ether methacrylate (or 2-[2-(2- methoxyethoxy)ethoxy] ethyl methacrylate) (TEGMA, 95%) was purified by an inhibitor remover (Aldrich #311332), under stirring for 5 min and then the inhibitors were removed by filtration. The initiator 2,2′-azobis(isobutylonitrile) (AIBN) was purified by recrystallization from methanol. Subsequently, 4-Cyano-4-[(dodecylsulfanylthiocarbonyl)sulfanyl]pentanol (CDTP), 4-(dimethylamino)pyridine (DMAP, ≥99%), 1,2-Dichloroethane (Cl_2_CH_2_CH_2_Cl_2_, anhydrous, 99.8%), and dichloromethane (CH_2_Cl_2_, anhydrous, ≥99.8%) were distilled under an argon atmosphere. All the abovementioned reagents and solvents were purchased from Sigma-Aldrich. Paclitaxel was purchased from Aurisco (Yangzhou, China). Regenerated cellulose dialysis membranes with molecular weight cut-off (MWCO) of 2 kDa were purchased from Spectra/Por. Pur-A-Lyzer Maxi 6000 dialysis microtubes with MWCO 6.0–8.0 kDa were purchased from Sigma-Aldrich (Prague, Czech Republic).

### 2.2. Size Exclusion Chromatography (SEC)

The molecular weights, i.e., number average molecular weight (*M_n_*), weight average molecular weight (*M_w_*) and the molecular weight distribution or dispersity (*Đ*) of the copolymers were determined by size exclusion chromatography (SEC). The SEC was performed on a setup equipped with PLgel 5 μm 100 Å and DeltaGel Mixed-B columns, an RI detector (watrex RI 200 Detector, Watrex Praha, s.r.o., Prague, Czech Republic), and chloroform–triethylamine–isopropanol (94/4/2 vol%) as the mobile phase at a flow rate of 1 mL·min^−1^. The molecular weight and dispersity values were based on polystyrene standards (PSS, Esslingen am Neckar, Germany).

### 2.3. NMR Spectroscopy

Temperature dependencies of ^1^H NMR spectra were recorded with Bruker Avance III 600 spectrometer operating at 600.2 MHz. The experimental parameters were as follows: 90° pulse width = 10 μs, acquisition time of 2.18 s, relaxation delay of 10 s, 16 scans. Before measurement, each solution was kept for 15 min at the desired temperature. The integrated intensities were determined with TopSpin 4.0.5 software with an accuracy of ±1%; 2D ^1^H–^1^H NOESY NMR spectra were acquired on the same instrument with 4098 Hz spectral window in f_1_ and f_2_ frequency axes. In total, 16 scans over 512 t_1_ (evolution time) increments with a relaxation delay of 10 s were accumulated. Mixing times used in measurements varied in the range of 100–1000 ms.

### 2.4. Dynamic Light Scattering (DLS) 

Dynamic light scattering (DLS) measurements were performed on a Malvern Zetasizer Nano ZS instrument (scattering angle: 173°) at a heating rate of ca. 0.4 °C min^−1^ with an interval of 2.0 °C; each temperature point was held for 10 min before measuring for equilibration of the sample. The samples were measured in water for injection (Ardeapharma, Ševětín, Czech Republic) at a concentration of 1 mg·mL^−1^. The averaged intensity autocorrelation functions were evaluated using the non-negative least squares (NNLS) analysis implemented in the Zetasizer software, resulting in a distribution of sizes converted from distributions of times using the Stokes–Einstein relation:

(1) $$R_{H}=\frac{k_{B}Tq^{2}}{6\pi \eta }\tau$$

where *k_B_* is the Boltzmann constant, *T* is the absolute temperature, *η* is the viscosity of the solvent, *τ* is the mean relaxation time related to the diffusion of the nanoparticles, and *q* is the scattering vector. *R_H_* is the hydrodynamic radius (2*R_H_* = diameter). 

### 2.5. Nanoparticles Preparation

The PLA-*b*-PTEGMA nanoparticles from the different BCs were prepared by using a nanoprecipitation protocol in water at 25 °C. A solution of BC was completely dissolved in acetone (1 mL, 5 mg·mL^−1^) and subsequently was added dropwise (EW-74900-00, Cole-Parmer^®^, Vernon Hills, IL, USA) to water for injection (4 mL) under stirring (250 rpm). Acetone was further removed by evaporation under reduced pressure at room temperature and the aqueous solution was concentrated to 2 mL. The prepared NPs were used immediately or stored at 4 °C. PTX-loaded BC3 nanoparticles were prepared following the same protocol, with the addition of 2 wt% of paclitaxel related to BC3, and the PTX concentration was measured using HPLC (see Section 2.6).

### 2.6. Paclitaxel Loading

The amount of chemotherapeutic paclitaxel loaded into the nanoparticles was measured by HPLC composed of a DIONEX UltiMate 3000 Pump and Diode Array Detector (UV-Vis) (Thermo Fisher Scientific, Waltham, MA, USA) by using a reverse-phase column Chromolith Performance RP-18e (100 × 4.6 mm, eluent water–acetonitrile with acetonitrile gradient 0–100 vol%, flow rate = 1.0 mL·min^−1^). Firstly, 10 μL of the PTX-loaded BC3 nanoparticles were collected from the bulk sample, filtered (0.45 μm), and diluted to 50 μL with acetonitrile. Afterwards, 20 μL of the final sample was injected through a sample loop. PTX was detected at 229 nm using ultraviolet (UV) detection. The retention time of PTX was 3.65 min in these experimental conditions. An analytical curve with a linear response in the range (0.5–100 μg·mL^−1^) was obtained and used to determine PTX contents.

The drug-loading content (LC) and the drug-loading efficiency (LE) were calculated by using the following equations:

(2) $$\mathrm{LC}\;\left(\%\right)=\frac{\mathrm{drug}\;\mathrm{amount}\;\mathrm{in}\;\mathrm{nanoparticles}}{\mathrm{mass}\;\mathrm{of}\;\mathrm{nanoparticles}}\;\times \;100$$

(3) $$\mathrm{LE}\;\left(\%\right)=\frac{\mathrm{drug}\;\mathrm{amount}\;\mathrm{in}\;\mathrm{nanoparticles}}{\mathrm{drug}\;\mathrm{feeding}}\;\times \;100$$

### 2.7. Drug Release Experiments

The paclitaxel release from the BC3 was studied in temperature-adjusted release media of 25, 37, and 40 °C adapted from previously published methodology [48]. In brief, aliquots (1 mL) of PTX-loaded BC3 NPs were loaded into Pur-A-Lyzer Maxi 6000 dialysis microtubes with MWCO 6.0–8.0 kDa. The microtubes were dialyzed against 3 L of water for injection with gentle stirring. The drug release experiments were performed in triplicate. At each sampling time, 10 µL was removed from the dialysis system and diluted to 50 μL by using acetonitrile. The PTX content at each sampling time was then determined via HPLC by applying the same procedure used to determine LC and LE.

### 2.8. In Vitro Stability

The stability of the NPs was monitored after placing them in contact with an RPMI culture medium supplemented with 10% *v*/*v* fetal bovine serum (FBS). Typically, 1 mg·mL^−1^ of the PTX-loaded BC3 NPs was placed in contact with the medium and the stability of NPs (NPs’ size and size distribution) was monitored by DLS for 24 h.

### 2.9. Transmission Electron Microscopy (TEM)

TEM observations were performed on a Tecnai G2 Spirit Twin at 120 kV (FEI, Brno, Czech Republic). The NPs were diluted 10-fold and used at 25 °C and 45 °C (after heating above LCST); 2 μL of each aqueous solution was dropped onto a copper TEM grid (300 mesh) coated with a thin, electron-transparent carbon film. Excess solution was removed by touching the bottom of the grid with filter paper. This rapid removal of the solution was performed after 1 min to minimize oversaturation during the drying process; this step was found to be necessary to preserve the structure of the NPs. The NPs were negatively stained with uranyl acetate (2 μL of a 1 wt% solution was dropped onto the dried NPs and removed after 15 s in the same manner described above). The samples were left to completely dry at ambient temperature and then observed via a TEM using bright field mode. Under these conditions, the micrographs displayed a negatively stained background with bright spots which correspond to the investigated NPs.

## 3. Results and Discussion

### 3.1. Synthesis of PLA-b-PTEGMA Block Copolymer via a One-Pot Protocol

The PLA-*b*-PTEGMA diblock copolymers were synthesized according to the literature [49]. It has been previously reported that the fine tuning of the LCST can be achieved by copolymerizing hydrophobics segments, for instance to OEGMA [44] and PNIPAM [50] homopolymers. In this way, a series of three PLA-*b*-PTEGMA diblock copolymers were prepared by the ROP-RAFT technique varying the monomers feed ratio towards an evaluation of the effect of the block hydrophobicity on the LCST. For this, the PLA molar ratio was varied from 0 to 80 mol% and a PTEGMA homopolymer was synthesized as a control (Table 1).

**Figure 1:**
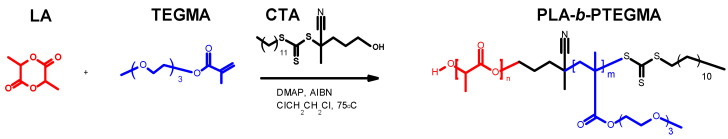
Scheme of synthesis of the PLA-*b*-PTEGMA block copolymer via one-pot ROP/RAFT polymerization. (LA = lactide; TEGMA = Triethylene glycol methyl ether methacrylate; CTA = chain transfer agent; PLA-*b*-PTEGMA = poly(lactide)-*b*-poly(triethylene glycol methyl ether methacrylate.)

**Figure 2:**
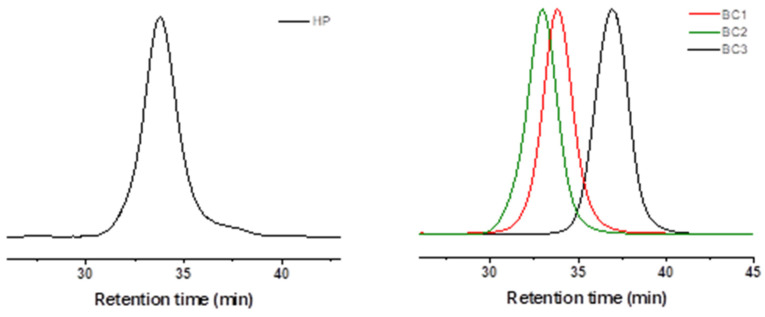
SEC chromatograms of the PTEGMA (**left**) and PLA-*b*-PTEGMA diblock copolymers (**right**) in chloroform–triethylamine–isopropanol (94/4/2 vol%).

Briefly, as an example for BC3, the feed ratio was as follows: [LA]:[CDTP]:[TEGMA]:[AIBN]:[DMAP] = [36]:[9]:[1]:[0.25]:[0.25]. LA (0.34 mg, 2.24 mmol), TEGMA (0.11 mL, 0.85 mmol), CDTP (0.05 g, 0.13 mmol), AIBN (0.006 g, 0.032 mmol), DMAP (0.63 mg, 0.52 mmol) and 0.75 mL of 1,2-dichloroethane were mixed in a dry 10 mL Schlenk flask. The reaction was conducted for 12 h under the argon atmosphere in a preheated oil bath at 75 °C. A typical simultaneously one-pot ROP of LA and RAFT of TEGMA is described (Figure 1): 

The conversion was calculated using ^1^H NMR spectra from the integral intensities of respective monomer and polymer signals. The final (co)polymers were purified by dialysis against acetone using a molecular weight cut-off (MWCO) of 2 kDa. The (co)polymers were recovered by evaporation of the solvent, dried under vacuum for about 24 h, and then analyzed by ^1^H NMR spectroscopy and SEC analysis. The ^1^H NMR spectra are shown in Figure S1a in Supplementary Materials, SEC chromatograms are presented in Figure 2, and results are summarized in Table 1. The subscripts of the homopolymers and copolymers refer to the weight average molecular weights of each block as determined by ^1^H NMR.

The SEC analysis of synthesized PLA-*b*-PTEGMA diblock copolymers clearly shows that the obtained curves are monomodal with rather symmetric elution peaks depicting a relatively narrow molecular weight distribution (Figure 2). The number average molecular weights, *M_n_*, and dispersity, *Ð*, provided in Table 1, together with time dependence plots of monomer conversion as well as ln([M]_0_/[M]_t_) (Figure S1b), suggest a well-controlled copolymerization.

### 3.2. Temperature Behavior: DLS, ^1^H NMR Spectra, and Fraction p of Proton Units (Groups) with Reduced Mobility

The temperature dependence behavior of the synthesized homo and block copolymers was studied in aqueous solutions using the combination of ^1^H NMR and DLS. The PTEGMA homopolymer is water-soluble and, therefore, was directly dissolved in an aqueous solution (water or D_2_O) and evaluated using ^1^H NMR and DLS as a function of temperature. However, the amphiphilic block copolymers containing the hydrophobic PLA blocks (BC1-BC3) are insoluble in water and require the preparation of NPs using the nanoprecipitation protocol. The technique consists of the rapid addition of the acetone-polymer solution into the aqueous solution (water for injection or D_2_O), which triggers the self-assembly of the polymer into NPs. The NPs prepared from the synthesized polymers using the nanoprecipitation technique (BC1 NPs, BC2 NPs, and BC3 NPs) were characterized as a function of temperature by ^1^H NMR and DLS; the results are presented below.

**Figure 3:**
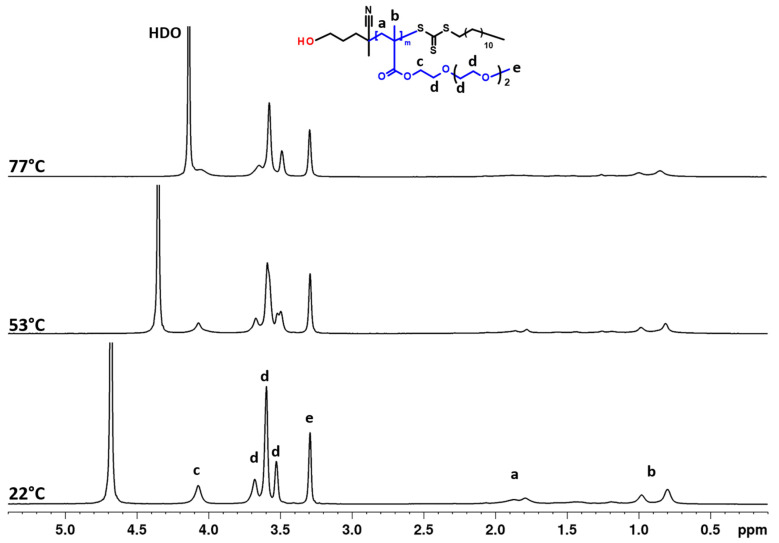
The 600.2 MHz ^1^H NMR spectra of PTEGMA homopolymer in D2O solution (c = 2.5 mg·mL^−1^) measured at 22, 53, and 77 °C under the same instrumental conditions.

**Figure 4:**
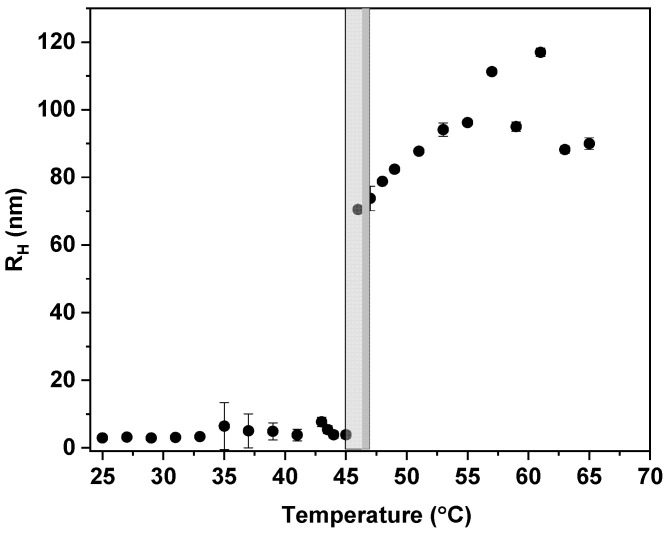
Hydrodynamic radius (*R_H_*) of PTEGMA homopolymer in water (c = 2.5 mg·mL^−1^) measured as a function of temperature.

High-resolution ^1^H NMR spectra of an aqueous solution (c = 2.5 mg·mL^−1^) of the PTEGMA homopolymer recorded under the same conditions at 3 temperatures—below the LCST (22 °C), in the middle of the transition (53 °C), and above the LCST (77 °C)—as well as the DLS measurements from 25 to 65 °C are presented in Figure 3 and Figure 4, respectively. The signals assignment of various types of proton measured at different temperatures is shown in the ^1^H NMR spectra and represented in the chemical structure of PTEGMA. Briefly, a signal from TEGMA side chain group C(O)OCH_2_, marked as “c”, was observed at δ ≈ 4.08 ppm, the peaks assigned as “d” (δ ≈ 3.80–3.40 ppm) correspond to OCH_2_ protons, and signal of the OCH_3_ group “e” is detected at *δ* = 3.29 ppm. Additionally, the broad signals “a” (δ ≈ 2.10–1.50 ppm) and “b” (δ ≈ 1 ppm) are related to methylene CH_2_ and methyl CH_3_ protons from the backbone of HP, respectively. At first glance, when comparing the spectra at different temperatures, there is a broadening and decrease of the intensity of polymer signals with increasing temperature. A closer observation of the “a, b” proton signals of polymer backbone as well as the resonance signals of “c” moiety showed that the disappearance of these signals is more pronounced in comparison to the signals of the side chain groups “d” and “e”. This suggests that mobility restriction is more significant close to and along the hydrophobic polymer backbone. Similar behavior was previously observed for PTEGMA homopolymer [31], PHPMA-*b*-PDEGMA, and POEGMA-based copolymers [44,51] and is in contrast to other thermo-responsive polymer-based systems, e.g., poly(*N*-isopropyl acrylamide) (PNIPAm) [52,53], poly(vinyl methyl ether) (PVME) [54], poly(*N*,*N*′-diethylacrylamide) [55], and PIPOx [56], in which all polymer signals were suppressed in the same way. The transition behavior is confirmed by the sudden increase in the intensity of the scattered light observed by DLS at the begging of the transition temperature (Figure S2). It indicates that the effect related to the decrease of the mobility of the polymer chains observed by ^1^H NMR with the increase of the temperature is due to the increase of the hydrophobicity of the HP chains and formation of polymer aggregates at around ~47 °C (Figure 4).

**Figure 5:**
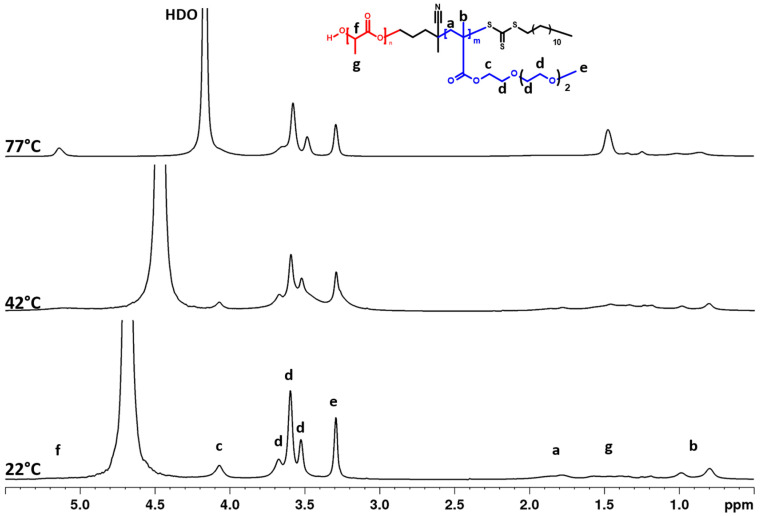
^1^H NMR spectra of PLA-*b*-PTEGMA (BC2) NPs in D_2_O solution (c = 2.8 mg·mL^−1^) measured at 22, 42, and 77 °C under the same instrumental conditions.

High-resolution ^1^H NMR spectra of prepared NPs (BC1, BC2, and BC3) and HP aqueous solutions measured at 22 °C are presented in Figure S3. There are two effects observed in the spectra in comparison to the spectra of respective BC1-BC3 copolymers in CDCl_3_ (see Figure S1). First, the signals of PLA are not observed in the spectra of the copolymers in the NP samples. This effect seems to be related to the structural features of the NPs produced by nanoprecipitation. The most likely NP structure produced by nanoprecipitation is related to the core-shell type and consists of NPs composed of a PLA core surrounded by the stabilizing corona of PTEGMA. In such a structural configuration, the mobility of PLA blocks is restricted within the hydrophobic, solid-like NPs’ core and, therefore, their signals appeared suppressed in the NMR spectra [57,58]. The second outcome observed on the spectra of the NPs is the effect of BC composition. It can be observed that the PTEGMA signals broaden with the increasing molar ratio of PLA in the copolymers (i.e., PTEGMA peaks in the spectrum of BC1 NPs with 14 mol% of PLA is very similar as for HP; however, the signals of PTEGMA are broader in the spectrum of BC3 NPs with 80 mol% of PLA). This seems to be related to the size ratio between the NPs core and corona. In BC3, the PTEGMA blocks are shorter in comparison to BC1 and BC2 and can physically interact with the PLA core. In order to evaluate the effect of PLA on PTEGMA phase transition temperature, the BC1-BC3 NPs solutions were measured in the temperature range of 22 to 77 °C. The high-resolution ^1^H NMR spectra of BC2 NP (only BC2 NPs for brevity) aqueous solution (c = 2.5 mg·mL^−1^) measured under the same instrumental conditions at 3 temperatures—below the LCST (22 °C), in the middle of the transition (42 °C), and above the LCST (77 °C)—are shown in Figure 5. The proton signals of PTEGMA block (“a, b, c, d, e”) are observed at similar positions as for HP (see Figure 3). In addition, similar behavior as observed for HP was observed for BC2: the decrease and broadening of integral intensities with the increase of temperature caused by decreasing the mobility of PTEGMA blocks. Interestingly, the opposite trend of the resonances was observed in the spectra for the PLA blocks (“f, g”). Their characteristic peaks start to appear and subsequently increase with the temperature rise. The same temperature effect on the PLA block was observed by NMR in NPs of PLA-*b*-PEO and PLA-*b*-Pluronics in aqueous solutions [57,58]. The appearance of PLA signals is most likely related to the increasing segmental motion of PLA chains with temperature elevation. There are three possibilities to explain this phenomenon: first is the weakening of the hydrophobic interactions between PLA chains due to crossing its glass transition temperature (*T_g_* of wet PLA is in the range of 26–39 °C); second, it can be caused by the presence of the thermo-responsive PTEGMA block (the dehydration of PTEGMA block above its LCST can cause the miscibility of the two blocks); a third possibility is the increase of the motion of the whole NPs [57,58,59].

**Figure 6:**
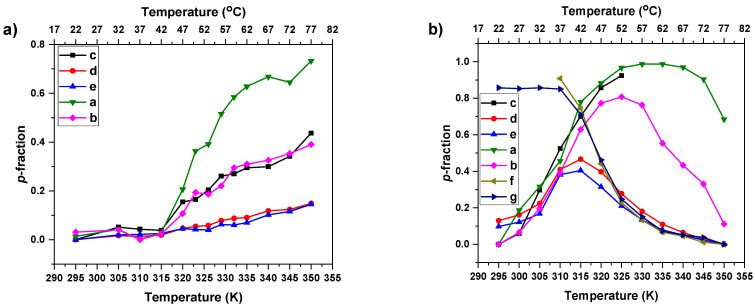
Temperature dependencies of the *p*- fraction as determined for signals of various proton types in aqueous solutions (c = 2.5 mg·mL^−1^) of HP (**a**) and BC2 (**b**) during gradual heating.

In the next step, we quantitatively characterized the changes occurring during the heating and cooling processes provided by the temperature-dependent integrated intensities of NMR signals. The values of the *p*-fraction of proton groups of the particular type with significantly reduced mobility were calculated according to the relation [51,56,59]:

(4) $$p=1-\frac{I\left(T\right)}{I\left(T_{0}\right)\times \frac{T_{0}}{T}}$$

where: *I(T)* is the integrated intensity of a particular polymer signal at related absolute temperature, *T*, and *I(T*_0_*)*, is the value of the integrated intensity of this signal when there is no phase separation (or other reason for the polymer segments’ mobility reduction). *T*_0_ was set for the temperature at which the integrated intensity of the particular signal was the highest (due to this, *p*(*T*_0_) = 0). Additionally, we consider that the integrated intensities should decrease with temperature as 1/*T* in the denominator of Equation (4). The temperature dependencies of the *p*-fraction of various proton types of HP in D_2_O solution (c = 2.5 mg·mL^−1^) are presented in Figure 6a. For all proton groups (signal assignment in Figure 3), *p*-fraction values remain unchanged until 42 °C. From 42 °C and above, the *p*-fraction values slowly start to increase, signaling the starting point of the phase transition. The increase of the *p*-fraction values determined from integrated intensities of various PTEGMA signals is dependent on the position (hydrophilicity) of the respective group in the polymer chain. In the main chain, the maximum value of the *p*-fraction (*p*_max_) of the hydrophobic group “a” (0.73) is almost twice that of the closest “b, c” moieties (*p*_max_ ≈ 0.4) and 5 times higher than the values of hydrophilic “d, e” groups (*p*_max_ ≈ 0.14). This indicates that the HP backbone is much more mobility-restricted (dehydrated) than the PTEGMA side chain. A similar effect was observed for PDEGMA and POEGMA-based copolymers solutions where the mobility of the backbone was also more affected by temperature in comparison to the side chains [44,51]. By using this approach, the LCST (defined as the temperature at *p*_max_/2) of the HP was estimated as 53 °C. The temperature behavior of the BC NPs was characterized in the same way, by measuring the temperature dependencies of their aqueous solutions. As an example, temperature dependencies of *p*-fraction of various proton types of BC2 NP solution (c = 2.5 mg·mL^−1^) are shown in Figure 6b. In this case, a different temperature behavior of PTEGMA proton groups is observed in comparison to HP (see Figure 6a). The *p*-fraction values start to grow from 22 °C and are divided into two types: one for the hydrophobic part of a chain “a, b, c” (main chain) with high *p*_max_ (>0.80) at 52 °C and the other for the hydrophilic side chain “d, e” with half lower *p*_max_ values (≈0.4) at 42 °C. However, independent of the values of *p*_max_ used for an estimation of the LCST (from the side or the main chain), the addition of the PLA block shifted the LCST of PTEGMA to lower temperature ranges between 32-37 °C. One interesting feature is that the values of *p*-fraction of PTEGMA decrease with a temperature above *p*_max_ points. This is an indication that the mobility of PTEGMA segments is increasing, which is unusual in comparison with the behavior observed for other thermo-responsive systems where the *p*-fraction value remains constant after reaching *p*_max_ [51,56,60]. 

This effect is related to the copolymer composition and became more pronounced with the increase of the molar ratio of PLA (see Figures S4 and S5). The starting values of the *p*-fraction of both PLA proton groups (“f and g” ≈ 0.9) start to decrease at 37 °C (LCST of PTEGMA main chain), reaching a minimum value at high temperature (77 °C). Moreover, this behavior also depends on the molar ratio between the PLA and PTEGMA blocks in the synthesized copolymer. In the case of BC1, the PLA (Figure S4) segments act as side-chain PTEGMA groups (somehow demonstrating the LCST behavior), but for BC3 NPs (Figure S5), the effect is almost the same as in the BC2 NPs sample. Similar behavior was observed for the κ-carrageenan where the decrease of the *p*-fraction with increasing temperature after LCST was attributed to the polymers UCST [60]. All the above-mentioned effects are related to different changes in the mobility of respective proton groups. It seems that at a certain point, with increasing temperature after LCST, the PTEGMA chains start to interact with the PLA chains leading to the increase in its overall mobility and in consequence the values of *p*-fraction.

**Figure 7:**
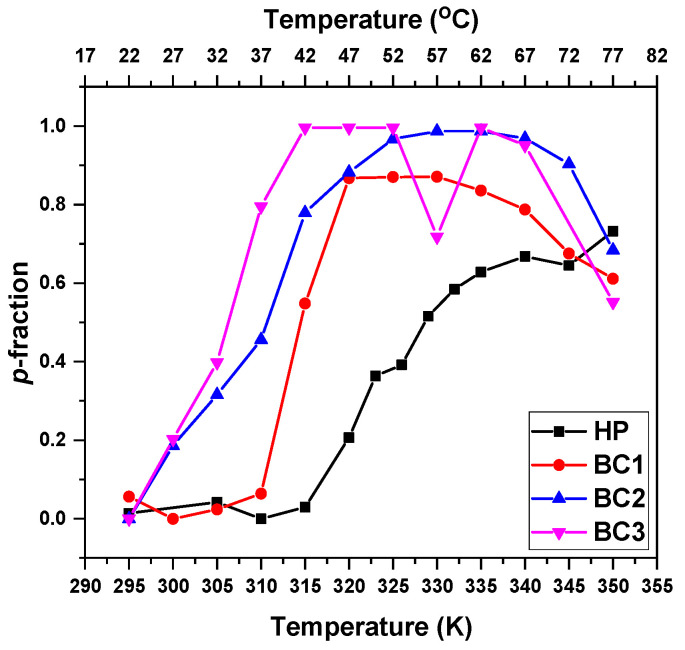
Temperature dependencies of the *p*-fraction for the PTEGMA CH_2_ protons (signal “a”) in D_2_O solutions (c = 2.5 mg·mL^−1^) of HP and BC1-BC3 copolymers determined during gradual heating.

For further comparisons, we chose the signal “a” from the PTEGMA backbone to characterize the effect of PLA on the LCST of the PTEGMA block. The temperature dependencies of the *p* fraction for all investigated solutions (c = 2.5 mg·mL^−1^) are presented in Figure 7. In comparison with the HP (LCST = 53 °C), the values of LCST for the block copolymers shifted to lower temperatures as a function of the PLA molar content in the copolymer following the relation: the higher the PLA content, the lower the LCST (BC1 = 42 °C, BC2 = 37 °C, and BC3 = 34 °C). The results are in good agreement with the data from the literature [61,62] as well as with the results from DLS. 

**Figure 8:**
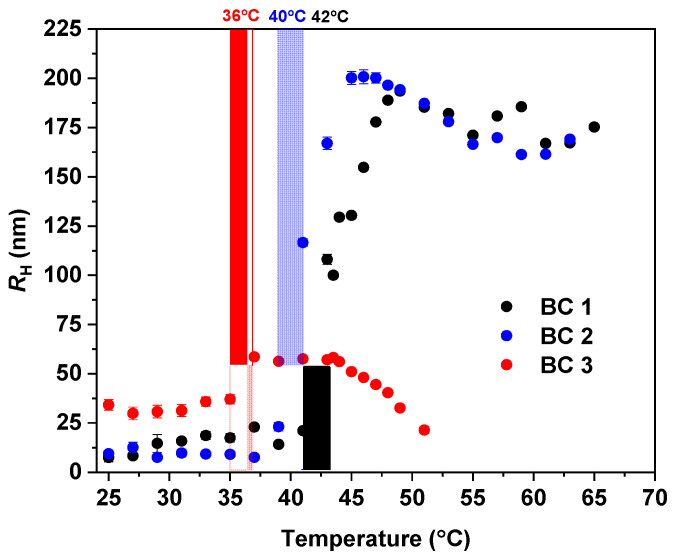
Hydrodynamic radius (*R_H_*) of the block copolymer NPs (BC1, BC2, and BC3) in water (c = 2.5 mg·mL^−1^) measured as a function of temperature.

The changes in the overall NPs’ scattering intensity (Figure S6), as well as the *R*_H_ (Figure 8) measured by DLS, confirm the trend observed by ^1^H NMR (Figure 7). As previously mentioned, by DLS the onset of the LCST is observed by the sudden increase of the scattering intensity caused by the NPs aggregation (also observed by TEM in Figure S7) due to the increase of their hydrophobicity with the increase in temperature. In comparison to the HP, the LCST of the NPs is shifted to 42, 40, and 36 °C for BC1, BC2, and BC3, respectively (Figure 8). The trend observed by both ^1^H NMR and DLS experiments clearly shows the influence of the PLA content on the LCST of the PTEGMA block. The higher the PLA content in the copolymer, the more hydrophobic and less hydrated are the resulting NPs and, consequently, the lower is their LCST. These results demonstrated once again the versatility of the addition of hydrophobic monomers such as PLA on the tuning of LCST temperatures of thermo-responsive polymers [62,63]. 

**Figure 9:**
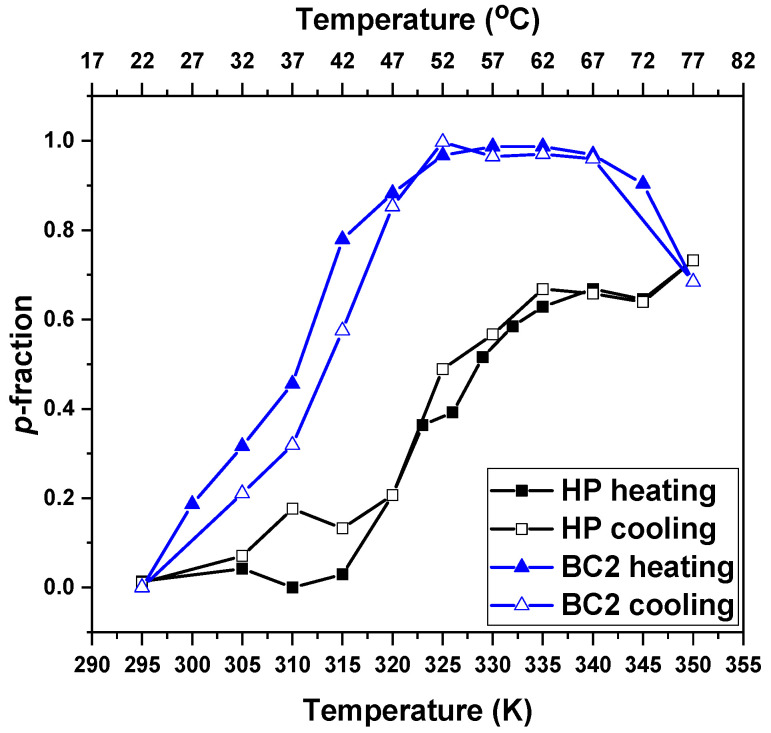
Temperature dependencies of the *p*- fraction for the PTEGMA CH_2_ protons (signal “a”) in D_2_O solutions (c = 2.5 mg·mL^−1^) of HP and BC2 NPs determined during gradual heating and subsequent gradual cooling.

In order to verify the reversibility of the LCST of the HP and block copolymer NPs, ^1^H NMR measurements were performed under gradual cooling right after the heating process. The results obtained for all proton types of respective solutions are presented in the Supplementary Material (Figures S8–S11). The temperature dependencies of the *p*-fraction from CH_2_ protons (“a”) of the PTEGMA in aqueous solutions (c = 2.5 mg·mL^−1^) from the samples of the HP and the BC2 during gradual heating and subsequent gradual cooling are shown in Figure 9. During the cooling process, all proton groups present similar behavior as that observed during heating. The LCST of the HP was observed at the same temperature, in agreement with the behavior of PDEGMA-based polymers reported previously [51]. However, for the block copolymer NPs, a shift of the LCST (of the PTEGMA) to higher temperature values was observed during the cooling process, which indicates that the vicinity of the PTEGMA chains within the NPs becomes more hydrophilic. The most probable reason for such behavior is that fewer PLA chains are in contact with PTEGMA chains in the NPs after the heating process, shifting the LCST temperature to higher values during the cooling process. Nevertheless, despite the shift in the LCST observed for the copolymer NP solutions during the cooling process, the overall results ascertained that the PTEGMA polymer and NP solutions exhibit reversible phase transition behavior.

### 3.3. 2D ^1^H–^1^H NOESY NMR Spectra: Conformational Changes of Polymers

**Figure 10:**
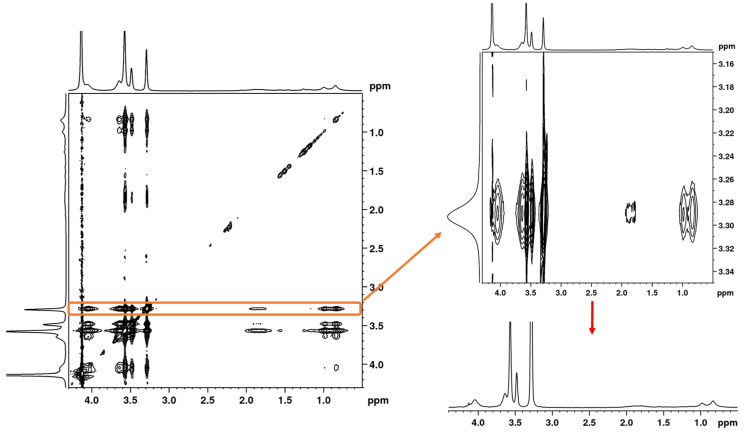
2D NOESY spectrum of HP in D_2_O solution (c = 2.5 mg·mL^−1^) measured 77 °C with mixing time 600 ms. At the top right: expanded part of the spectrum close to PTEGMA “e” group cross-peaks; at the bottom right: 1D slice spectrum extracted from the PTEMA “e” signal of the NOESY spectrum.

**Figure 11:**
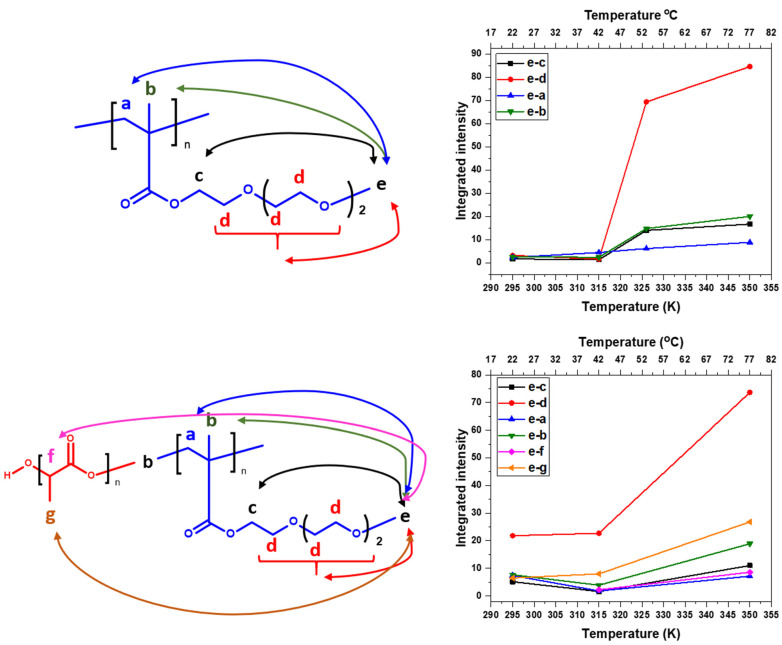
Chemical structures and intermolecular correlations in HP (**upper left**) and BC2 (**lower left**). Temperature dependencies of integrated intensities of various signals in 1D slices extracted from the signal of “e” protons of PTEGMA units of the 2D NOESY of D_2_O solutions (c = 2.5 mg·mL^−1^) of the HP (**upper right**) and BC2 (**lower right**).

In order to better describe the changes occurring during the phase transition, 2D nuclear Overhauser effect spectroscopy (NOESY) was used to obtain information on spatial proximity between proton groups of PTEGMA and PLA-*b*-PTEGMA units. By measuring NOESY NMR experiments, it is possible to obtain information on the spatial interactions of different nuclear spins which are at a maximum distance of 0.5 nm [12,45,51,64]. We chose the HP and the BC2 NP aqueous solutions to compare the differences between homo-and copolymers. The 2D ^1^H–^1^H NOESY NMR spectra were measured at selected temperature points of interest (i.e., in the case of HP, at 22 °C, at 42 °C right below LCST, at 52 °C, which is at the LCST, and at 77 °C which is above LCST). Due to its low *p*_max_, as well as good detection in the high-resolution NMR spectra, the OCH_3_ group (“e”) from PTEGMA was chosen for further consideration. For quantitative characterization, 1D slices from 2D NOESY spectra were used (see Figure 10). Surprisingly, in the NOESY spectrum of HP, measured at 22 °C, a weak cross-peak between group “e” and other proton groups of PTEGMA units was detected, suggesting that PTEGMA chains are in a hydrated state in a random-coil conformation with predominant polymer—water interactions. The same configuration was observed at the temperature below the transition (42 °C). Further heating of the solution resulted in an increase of the integral intensity of all cross-peaks (see Figure 11), which indicates that the number of contacts between the respective proton groups at distances smaller than 0.5 nm increased. These contacts can be from the groups of the same chains of the homopolymer (intramolecular) or between individual chains (intermolecular). In the middle of the transition, at the temperature of 52 °C, the polymer-water interactions are replaced by polymer–polymer interactions, which indicates the formation of globular structures (aggregates). The same approach was used for the study of the BC2 NPs (see Figures S12 and S11). In comparison with the HP solution at 22 °C, the cross-peaks detected between groups “e” and “d” have higher intensities, which is reasonable, since the PTEGMA units are in closer contact at the NPs corona. At 42 °C (temperature of maximum *p*-fraction of “e” group), the intensities of the cross-peaks do not change, most likely because at this temperature, the signals of all PTEGMA groups have higher values of *p*_max_ (reported in Figure 6b) and the detection produced corresponds to the interactions between “still mobile” groups. However, at 77 °C the integral intensities of all cross-peaks increase, especially the “e–g” groups (cross-peak between group “e” of PTEGMA and methyl group “g” of PLA). This suggests that at higher temperatures, the number of contacts between the core and corona increases. This seems to be caused by NP aggregation as well as core-corona inner particle structural changes. These findings, together with the values of the *p*-fraction mentioned above, confirm the effect of the PLA block on PTEGMA temperature behavior.

### 3.4. Paclitaxel-Loaded BC3 Nanoparticles and Paclitaxel Release

**Figure 12:**
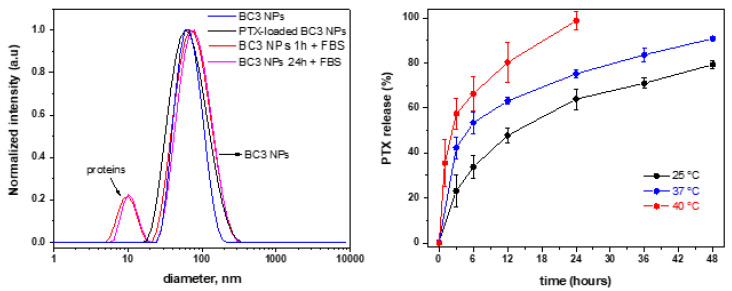
Size distributions obtained by DLS for PTX-unloaded BC3 NPs (blue lines), PTX-loaded BC3 NPs (black lines), and in the presence of 10% FBS after 1 h (red lines) and 24 h (magenta lines) (**left**), in addition to drug release profiles from PTX-loaded BC3 at 25 (black lines), 37 (blue lines), and 40 °C (red lines) (**right**).

According to NMR and DLS results, BC3 demonstrated the most relevant LCST transitions appropriate to application as a drug delivery system (LCST ~34–36 °C) [18]. Next, we manufactured BC3 NPs loaded with the chemotherapeutic drug paclitaxel, because of its hydrophobicity and wide range of applications in chemotherapeutic treatment. For example, a paclitaxel formulation, Cremophor^®^ EL (CrEL), was a first-line treatment for breast, lung, and ovarian cancer, and a second-line treatment for Kaposi sarcoma in HIV patients for many years [65]. However, because of its poor water solubility, PTX in CrEL is formulated together with low-molecular-weight surfactant (castor bean oil by PEGylation), exhibiting dose-related side effects in humans. To overcome dose-related side effects, PTX was formulated as NPs manufactured from block copolymers such as PLA-*b*-PEO, e.g., Genexol-PM^®^ [66]. These PLA_1.75k_-*b*-PEO_2k_ formulation-containing paclitaxels was first approved in Korea (2007) and become the first block copolymer NP formulation product approved based on PTX [67,68,69]. Taking into consideration the similarities between the block copolymer of Genexol-PM and BC3, such as molecular weight and block copolymer chemistry (Table 1), we then further evaluated the potential application of BC3 as a drug-delivery system. Firstly, the stability of the BC3 NPs was monitored by evaluating their diameter size in serum as a function of time. The left of Figure 12 (red and magenta lines) shows the temporal stability of the BC3 in 10% (v/v) diluted FBS in RPMI medium as a function of the incubation time. The size patterns do not change in 24 h suggesting that the NPs are highly stable against aggregation in the simulated physiological media (Figure 12, left, magenta lines) [70]. The slight increase in diameter (~8–10 nm) is generally attributed to the adsorption of a protein monolayer (corona), because the average size of the dissolved single proteins is ≈8 nm [71,72], as evidenced by the small peak observed at the region of ~10 nm diameter (Figure 12, red and magenta lines).

Because we previously demonstrated that the stability of PTX-loaded PLA NPs is limited to a ~2–3% *w*_drug_/*w*_polymer_ drug feeding by nanoprecipitation [48], we selected a 2% *w*_drug_/*w*_polymer_ drug feeding for this experiment. Monodisperse PTX-loaded BC3 NPs were prepared by the nanoprecipitation protocol comprising NPs with ~65 nm in diameter and polydispersity of 0.167 (Figure 12, left, black lines), with a similar diameter to the PTX-unloaded BC3 NPs (Figure 12, left–blue lines; ~62 nm in diameter and polydispersity of 0.135). The LC was 1.6% ± 0.2 *w*_drug_/*w*_polymer_ with a LE of ~81 ± 2.5%, reasonably reproducible, and nearly constant for the 3 batches calculated using Equations (2) and (3), respectively. The drug release profiles at a temperature of 25, 37, and 40 °C were monitored by HPLC and the results are shown in Figure 12, on the right side.

It is well known that drug release is generally governed by various mechanisms, such as by drug diffusion-controlled release, by the trigger initiated after changes in the environmental conditions, i.e., the decrease of pH, by redox conditions, or by the increase of temperature or bulk erosion rate, when considering nanoparticles produced from biodegradable polymers [73,74].

Figure 12, on the right, provides evidence that the drug release profile is clearly temperature-dependent. The paclitaxel release is sustained in all tested conditions; however, it is substantially accelerated at temperatures of 37 (>90% released in 48 h) and 40 °C (>90% released in 24 h) when compared to 25 °C (>80% released in 48 h). These results suggest that at temperatures closer to the LCST (37 °C), the BC3 NPs start to become physically destabilized (temperature-triggered activation–de-swelling aggregation) [75,76], thus accelerating the release of the anticancer drug. This effect is most pronounced at a temperature above the LCST, where the PTX is released completely within the first 24 h at 40 °C, mimicking, for instance, temperatures in the range used for hyperthermia treatment [46,77].

The results above strongly suggest that the PTX-loaded BC3 NPs based on PLA_2.6K_-*b*-PTEGMA_2K_ thermo-responsive block copolymer exhibit physicochemical properties required for potential application as drug delivery systems via temperature-triggered release mechanism. This is so because, considering their size (~65 nm) and stability in simulated physiological conditions (Figure 12, left, magenta lines), the BC3 NPs could potentially be accumulated in solid tumor sites via the well-known EPR effect [78]. In addition, the nanostructures are capable of forming cargo hydrophobic guest molecules into their core during the circulation time and the cargo content can be selectively and quickly accelerated with the addition of heat, for example, in the hyperthermia treatment.

## 4. Conclusions

In this work, amphiphilic, biocompatible, and thermo-teresponsive poly(lactide)-*b*- poly(triethylene glycol methyl ether methacrylate) (PLA-*b*-PTEGMA) block copolymers were successfully synthesized via a combination of ring-opening polymerization (ROP) and reversible addition–fragmentation chain transfer (RAFT) polymerization (ROP-RAFT) and characterized by using SEC and NMR techniques. The copolymers were synthesized with different molecular weights and molar fractions of the PLA block with good control over their characteristics, conferring the capability to fine tune the LCST accordingly to the length of the PLA and PTEGMA blocks. These features were studied in solutions of NPs manufactured from the different blocks by using ^1^H NMR spectroscopy and NOESY in combination with DLS as a function of temperature changes. It was observed that the LCST of the PLA-*b*-PTEGMA copolymers shift to lower temperatures with the increase of the molar fraction of the PLA in the copolymers, for example, from 53 °C (0 mol% of PLA), 42 °C (14 mol% of PLA), or 40 °C (59 mol% of PLA), to 36 °C (80 mol% of PLA), which clearly indicates the effect of the hydrophobicity of the PLA block on the LCST of the PTEGMA block. PTX-loaded NPs were successfully manufactured, from the most hydrophobic block copolymer, as well as from that which presents LCST transitions at physiologically relevant temperatures (38–45 °C), for the subsequent study of the PTX encapsulation-drug-release. The chemotherapeutic release profiles were clearly temperature-dependent, with the PTX release sustained in all tested conditions; however, this was substantially accelerated at temperatures of 37 and 40 °C when compared to 25 °C. Further stability studies of the NPs in physiological simulated conditions and their size (~65 nm), together with their capability of PTX encapsulation and release via a temperature-triggered drug release mechanism, make PLA-*b*-PTEGMA copolymers suitable candidates for applications in biomedicine and/or drug and gene delivery systems. 

## Figures and Tables

**Table 1:** Molecular characteristics of the PTEGMA homopolymer and PLA-*b*-PTEGMA block copolymers.

Sample	LA/TEGMA Feed Ratio	Conv. ^a^ (%) LA/TEGMA	PLA/TEGMA NMR ^b^	*M*_n_, ^1^H NMR [g·mol^−1^] ^c^	*M_n_*, SEC ^d^ [g·mol^−1^]	*Ð* ^d^
PTEGMA_14.2K_ (HP)	0/65	0/94	0/100	14,200	14,600	1.16
PLA_0.86K_-*b*-PTEGMA_14.5K_ (BC1)	12/65	88/96	14/86	15,400	14,700	1.16
PLA_5.2K_-*b*-PTEGMA_9.5K_ (BC2)	70/43	96/95	59/41	14,800	14,300	1.21
PLA_2.6K_-*b*-PTEGMA_2K_ (BC3)	36/9	92/94	80/20	4600	6200	1.13

^a^ Determined by ^1^H NMR in CDCl_3_. ^b^ Molar ratio in the copolymer determined by ^1^H NMR spectra. ^c^ Determined by ^1^H NMR according to conversion. ^d^ Determined by SEC in chloroform–triethylamine–isopropanol (94/4/2 vol%).

## Data Availability

The data presented in this study are available on request from the corresponding author.

## Supplementary Materials

The following supporting information can be downloaded at: https://www.mdpi.com/article/10.3390/pharmaceutics15041191/s1, Figure S1a. ^1^H NMR spectra of PTEGMA homopolymer (HP) and PLA-*b*-PTEGMA block copolymers (BC1-BC3) measured in CDCl_3_ at 22 °C. Figure S1b. Conversions of LA and TEGMA versus polymerization time (A) and ln( [M]_0_/[M]_t_) of LA and TEGMA versus polymerization time (B). Figure S2. Scattering intensity of PTEGMA homopolymer in water (c = 2.5 mg·mL^−1^) measured by DLS as a function of temperature at heating rate of 0.4 °C·min^−1^. Figure S3. ^1^H NMR spectra of PTEGMA homopolymer (HP) and PLA-*b*-PTEGMA block copolymers (BC1-BC3) NPs measured in D_2_O at 22 °C. Figure S4. Temperature dependencies of the *p*-fraction as determined for signals of various proton types in aqueous solution (c = 2.5 mg·mL^−1^) of BC1 NPs during gradual heating. Figure S5. Temperature dependencies of the *p*-fraction as determined for signals of various proton types in aqueous solution (c = 2.5 mg·mL^−1^) of BC3 NPs during gradual heating. Figure S6. Scattering intensity of NP samples in water (c = 2.5 mg·mL^1^) measured by DLS as a function of temperature at heating rate of 0.4 °C/min. Figure S7. TEM images of BC3 NPs measured below (left) and above (right) LCST. Figure S8. Temperature dependencies of the *p*-fraction as determined for signals of various proton types in aqueous solution (c = 2.5 mg·mL^−1^) of HP during gradual cooling. Figure S9. Temperature dependencies of the *p*-fraction as determined for signals of various proton types in aqueous solution (c = 2.5 mg·mL^−1^) of BC1 NPs during gradual cooling. Figure S10. Temperature dependencies of the *p*-fraction as determined for signals of various proton types in aqueous solution (c = 2.5 mg·mL^−1^) of BC2 NPs during gradual cooling. Figure S11. Temperature dependencies of the *p*- fraction as determined for signals of various proton types in aqueous solution (c = 2.5 mg·mL^−1^) of BC3 NPs during gradual cooling. Figure S12. 2D NOESY spectrum of BC2 NPs in D2O solution (c = 2.5 mg·mL^−1^) measured 77 °C with mixing time 300 ms. At the upper right, there is an expanded part of the spectrum close to PTEGMA “e” group cross-peaks, at the lower right there is an 1D slice spectrum extracted from the PTEGMA “e” signal of the NOESY spectrum.
