# Supplementary material for: Smart Poly(lactide)-b-poly(triethylene glycol methyl ether methacrylate) (PLA-b-PTEGMA) Block Copolymers: One-Pot Synthesis, Temperature Behavior, and Controlled Release of Paclitaxel
Source: Pharmaceutics. 2023 Apr 8;15(4):1191. doi: 10.3390/pharmaceutics15041191 (PMC10143907; doi:10.3390/pharmaceutics15041191)
Supplement: Supplementary file 1 [file pharmaceutics-15-01191-s001.zip › pharmaceutics-2301364-supplementary.pdf]

# Supplementary Materials

# Smart Poly(lactide)-*b*-poly(triethylene glycol methyl ether methacrylate) (PLA-*b*-PTEGMA) Block Copolymers: One-Pot Synthesis, Temperature Behavior, and Controlled Release of Paclitaxel

**Svetlana Lukáš Petrova, Martina Vragović, Ewa Pavlova, Zulfiya Černochová, Alessandro Jäger, Eliézer Jäger and Rafał Konefał ***

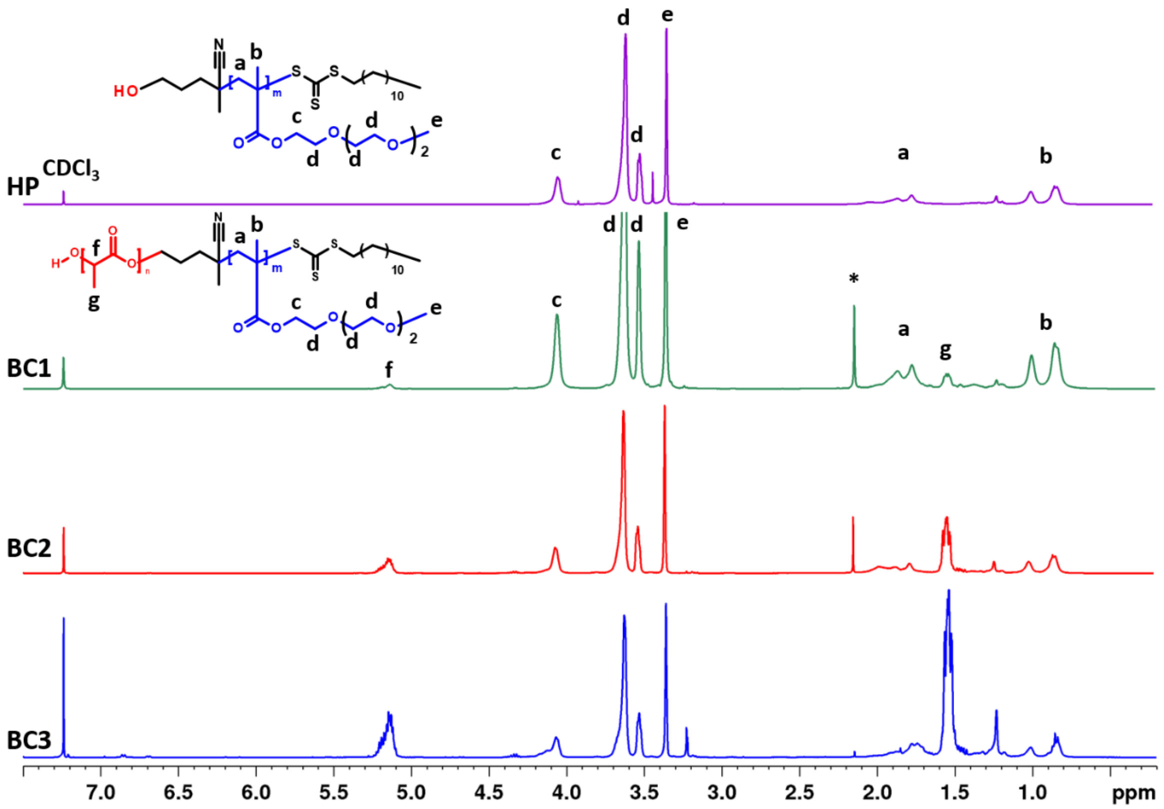


**Figure S1a.** $^{1}$H NMR spectra of PTEGMA homopolymer (HP) and PLA-*b*-PTEGMA block copolymers (BC1-BC3) measured in $CDCl_3$ at 22 °C.

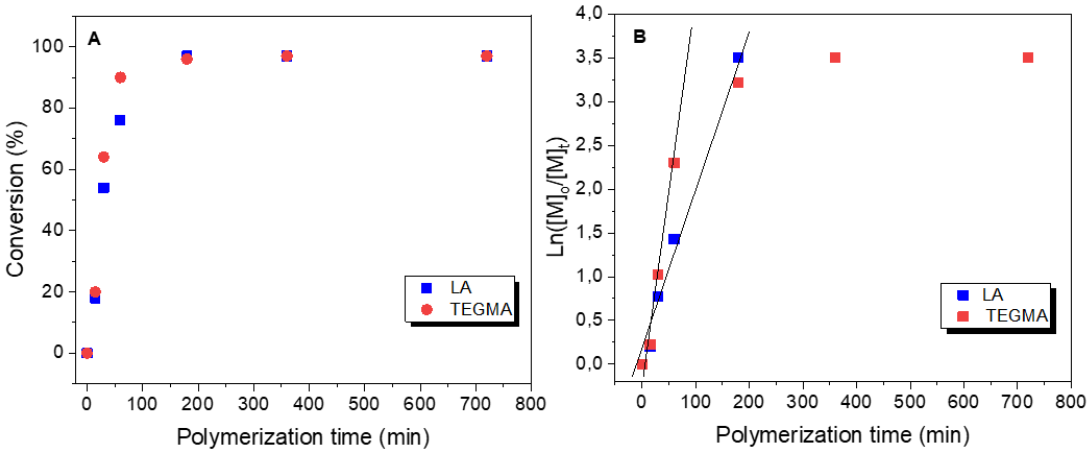


**Figure S1b.** Conversions of LA and TEGMA versus polymerization time(A) and ln($[M]_0/[M]_t$) of LA and TEGMA versus polymerization time (B)

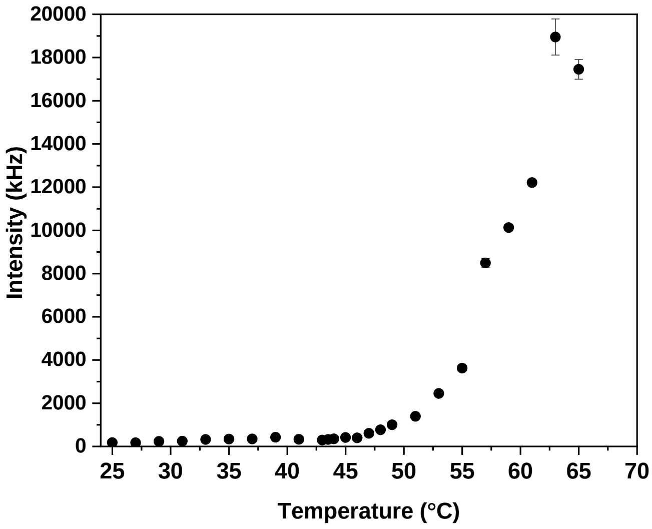


**Figure S2**. Scattering intensity of PTEGMA homopolymer in water (c = 2.5 mg·ml$^{-1}$) measured by DLS as a function of temperature at heating rate of of 0.4 °C·min$^{-1}$.

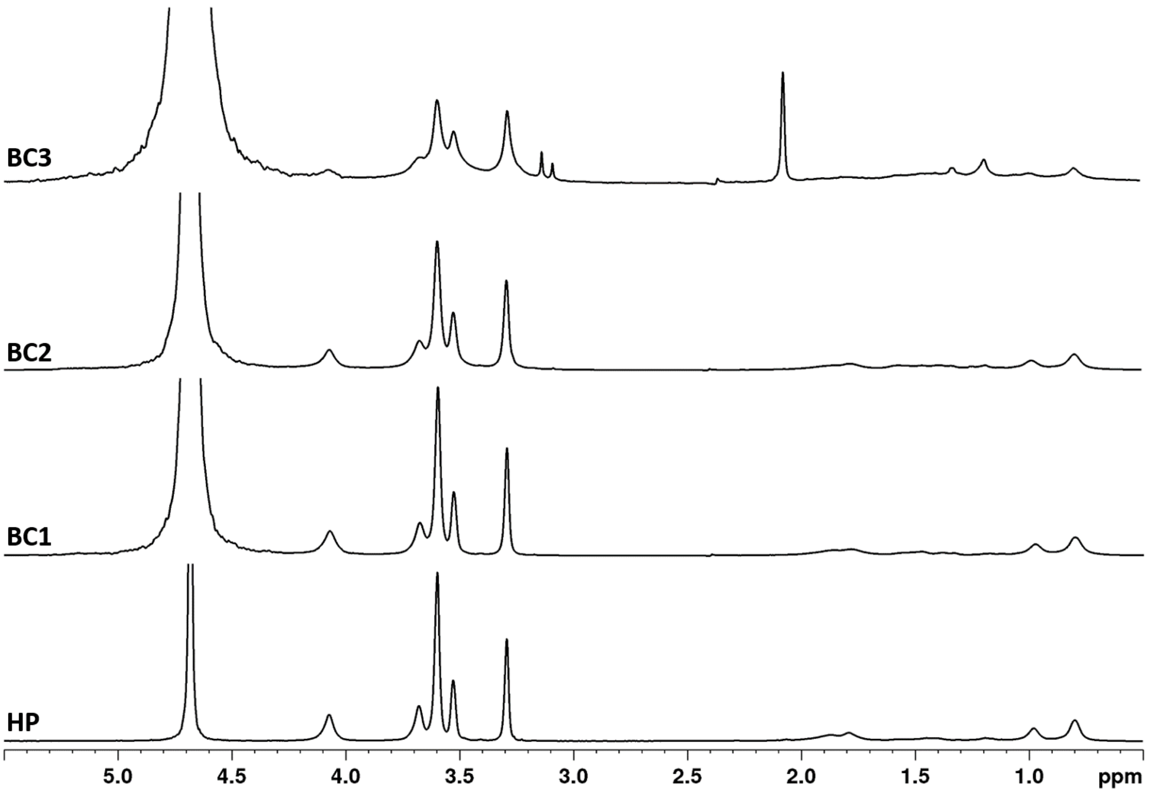


**Figure S3.** $^{1}H$ NMR spectra of PTEGMA homopolymer (HP) and PLA-*b*-PTEGMA block copolymers (BC1-BC3) NPs measured in $D_2O$ at 22°C.

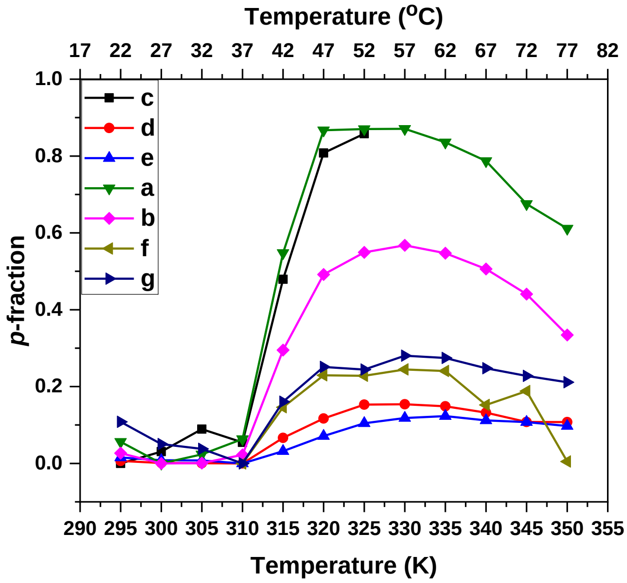


**Figure S4.** Temperature dependences of the *p*- fraction as determined for signals of various proton types in aqueous solution (c = 2.5 mg·mL$^{-1}$) of BC1 NPs during gradual heating.

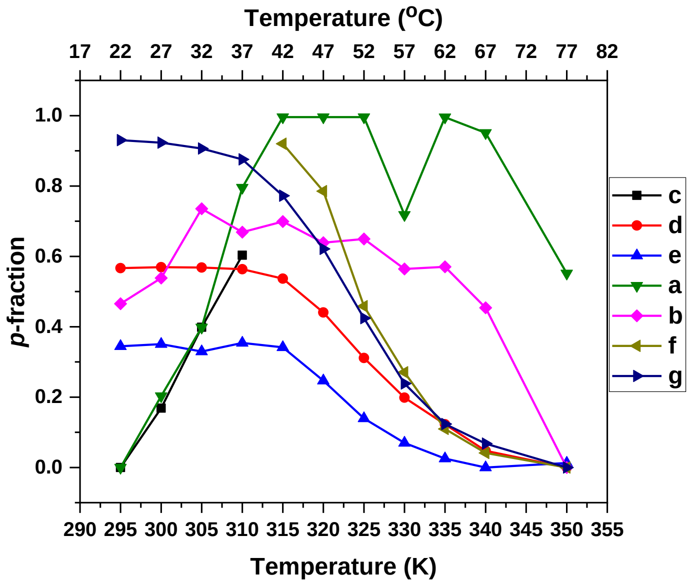


**Figure S5.** Temperature dependences of the *p*- fraction as determined for signals of various proton types in aqueous solution (c = 2.5 mg·mL[-1]) of BC3 NPs during gradual heating.

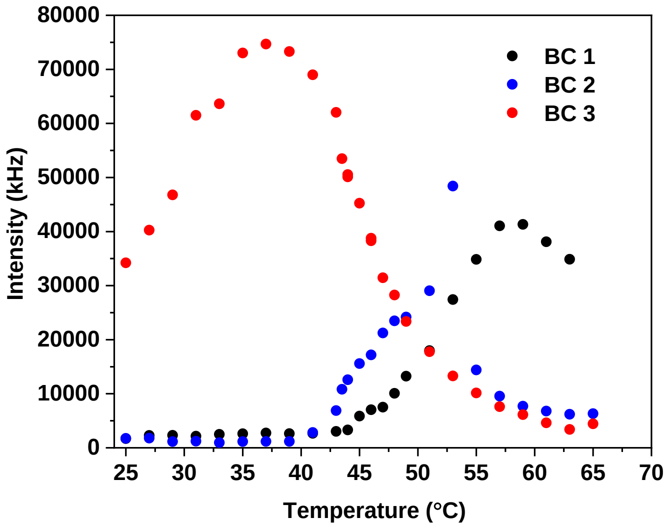


**Figure S6**. Scattering intensity of NPs samples in water (c = 2.5 mg·mL[1]) measured by DLS as a function of temperature at heating rate of of 0.4 °C /min.

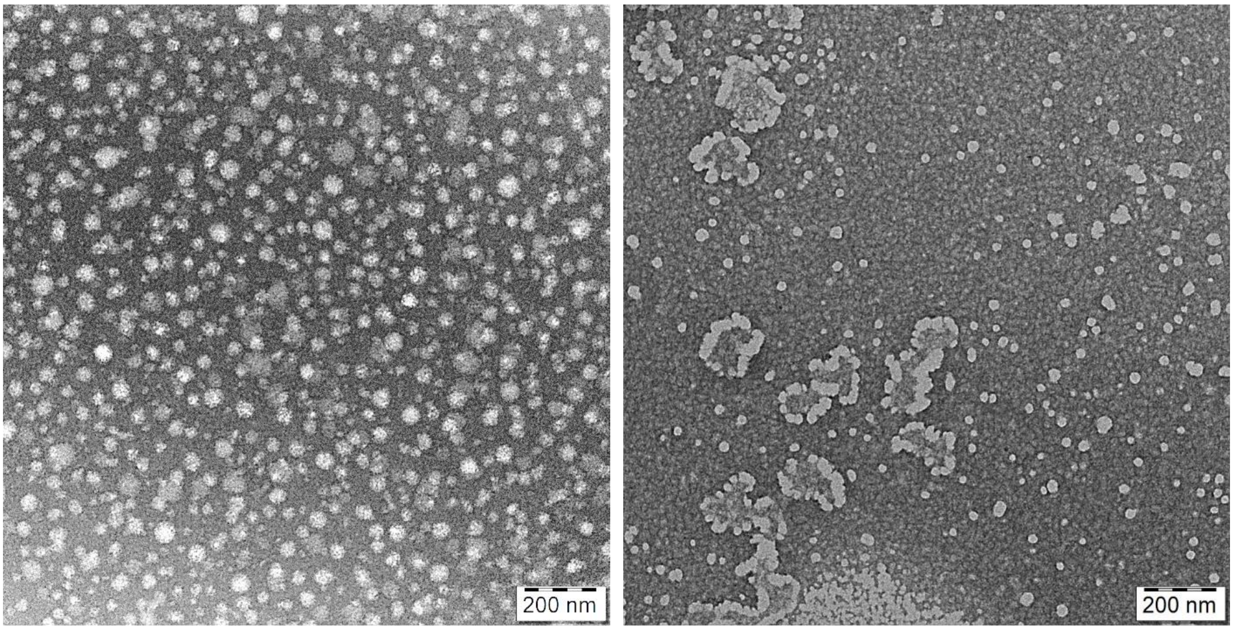


**Figure S7.** TEM images of BC3 NPs measured below (left) and above (right) LCST.

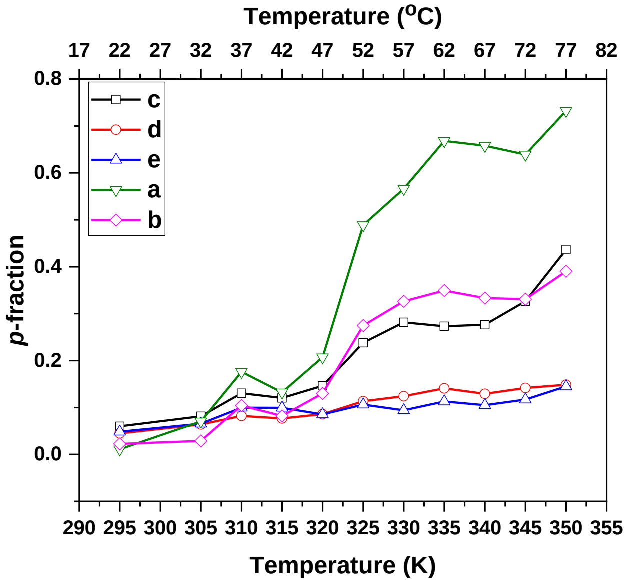


**Figure S8.** Temperature dependences of the *p*- fraction as determined for signals of various proton types in aqueous solution (c = 2.5 mg·mL$^{-1}$) of HP during gradual cooling.

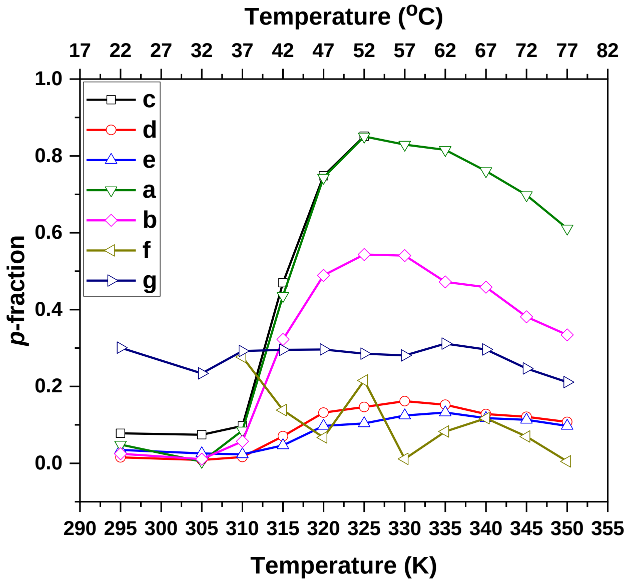


**Figure S9.** Temperature dependences of the *p*- fraction as determined for signals of various proton types in aqueous solution (c = 2.5 mg·mL$^{-1}$) of BC1 NPs during gradual cooling.

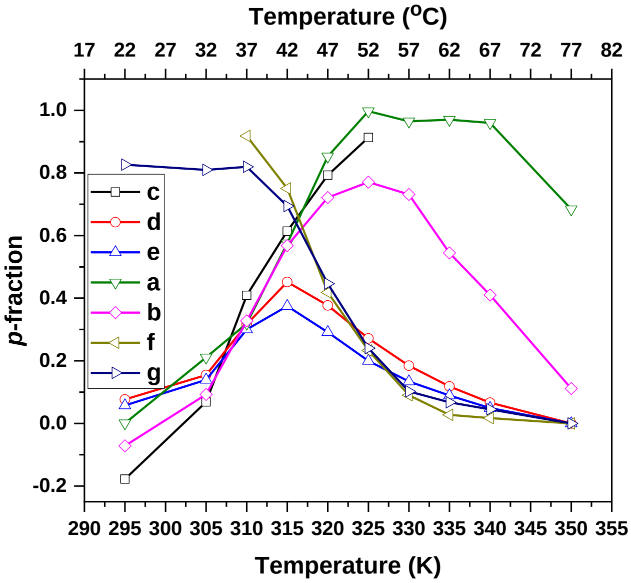


**Figure S10.** Temperature dependences of the *p*- fraction as determined for signals of various proton types in aqueous solution (c = 2.5 mg·mL$^{-1}$) of BC2 NPs during gradual cooling.

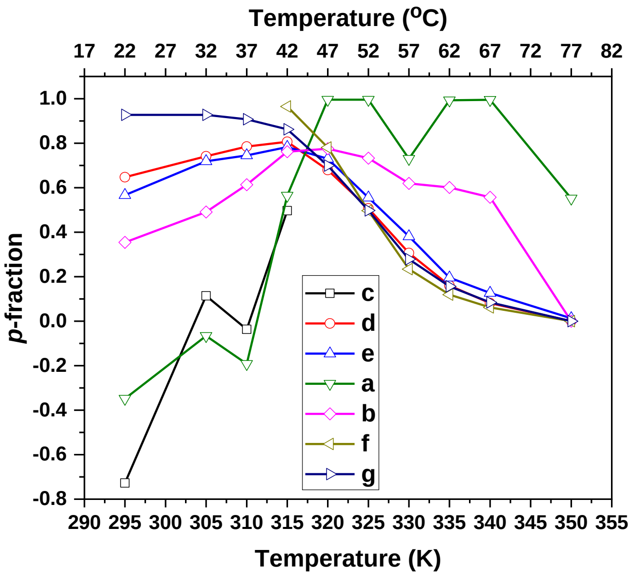


**Figure S11.** Temperature dependences of the *p*- fraction as determined for signals of various proton types in aqueous solution (c = 2.5 mg·mL$^{-1}$) of BC3 NPs during gradual cooling.

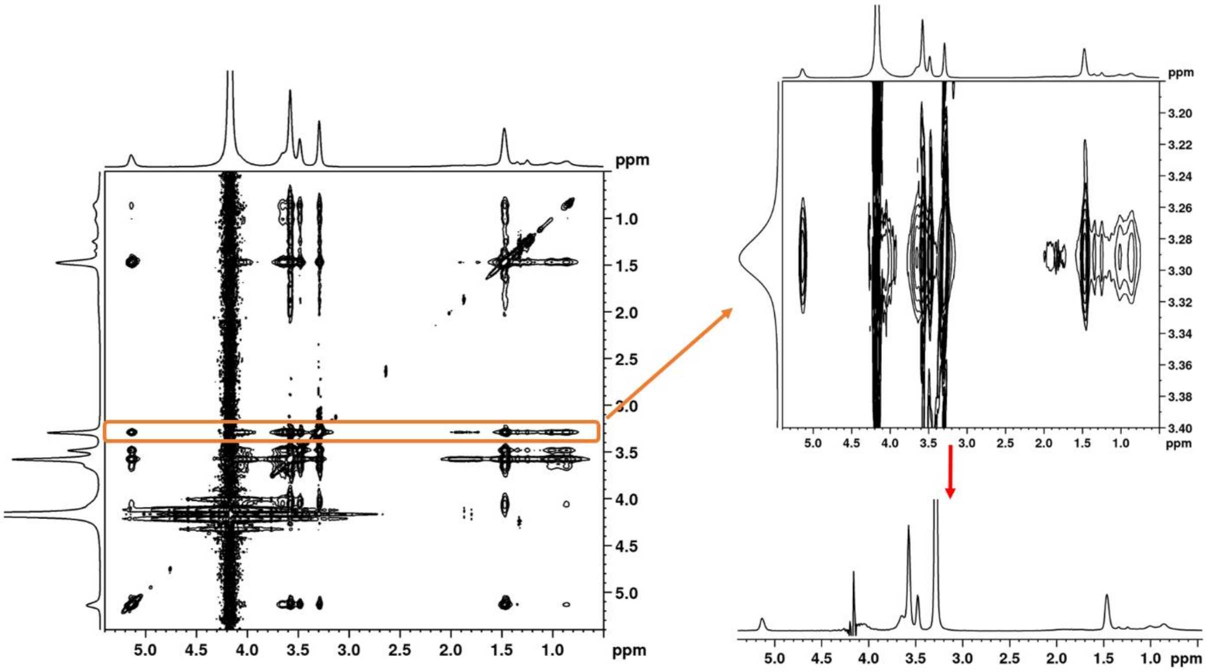


**Figure S12.** 2D NOESY spectrum of BC2 NPs in D2O solution (c = 2.5 mg·mL$^{-1}$) measured 77 °C with mixing time 300 ms. On the right up there is expanded part of the spectrum close to PTEGMA "e" group cross-peaks, on the right down there is 1D slice spectrum extracted from the PTEGMA "e" signal of the NOESY spectrum.
